# Switching Tenofovir/Emtricitabine plus Lopinavir/r to Raltegravir plus Darunavir/r in Patients with Suppressed Viral Load Did Not Result in Improvement of Renal Function but Could Sustain Viral Suppression: A Randomized Multicenter Trial

**DOI:** 10.1371/journal.pone.0073639

**Published:** 2013-08-08

**Authors:** Takeshi Nishijima, Hiroyuki Gatanaga, Takuro Shimbo, Hirokazu Komatsu, Tomoyuki Endo, Masahide Horiba, Michiko Koga, Toshio Naito, Ichiro Itoda, Masanori Tei, Teruhisa Fujii, Kiyonori Takada, Masahiro Yamamoto, Toshikazu Miyakawa, Yoshinari Tanabe, Hiroaki Mitsuya, Shinichi Oka, on behalf of the SPARE study team

**Affiliations:** 1 AIDS Clinical Center, National Center for Global Health and Medicine, Tokyo, Japan; 2 Center for AIDS Research, Kumamoto University School of Medicine, Kumamoto, Japan; 3 Department of Clinical Study and Informatics, Center for Clinical Sciences, National Center for Global Health and Medicine, Tokyo, Japan; 4 Department of Community Care, Saku Central Hospital, Nagano, Japan; 5 Department of Hematology, Hokkaido University Hospital, Sapporo, Japan; 6 Division of Respiratory Medicine, Higashisaitama National Hospital, Saitama, Japan; 7 Division of Infectious Diseases, Advanced Clinical Research Center, the Institute of Medical Science, University of Tokyo, Tokyo, Japan; 8 Department of General Medicine, Juntendo University School of Medicine, Tokyo, Japan; 9 Shirakaba Clinic, Tokyo, Japan; 10 Department of Integrated Medicine, Saku Central Hospital, Nagano, Japan; 11 Division of Blood Transfusion, Hiroshima University Hospital, Hiroshima, Japan; 12 Postgraduate Clinical Training Center, Ehime University Hospital, Ehime, Japan; 13 Internal Medicine, Clinical Research Institute, National Hospital Organization Kyushu Medical Center, Fukuoka, Japan; 14 Department of Infectious Diseases and Hematology, Kumamoto University School of Medicine, Kumamoto, Japan; 15 Division of Infection Control and Prevention, Niigata University Medical and Dental Hospital, Niigata, Japan; 16 Experimental Retrovirology Section, HIV and AIDS Malignancy Branch, National Cancer Institute, National Institutes of Health, Bethesda, Maryland, United States of America; University of Ottawa, Canada

## Abstract

**Background:**

Whether tenofovir nephrotoxicity is reversible after its withdrawal is unknown. Furthermore, there are no data on the viral efficacy of raltegravir (RAL) plus ritonavir-boosted Darunavir (DRV/r) in patients with suppressed viral load.

**Methods:**

This multicenter, randomized trial compared renal function and viral efficacy in patients with suppressed viral load treated with RAL+DRV/r and ritonavir-boosted lopinavir (LPV/r) plus tenofovir/emtricitabine (TVD), who had been previously on LPV/r+TVD. The primary endpoint was the proportion of patients with >10% improvement in estimated glomerular filtration rate (eGFR) at 48 weeks calculated with Cockcroft-Gault equation.

**Results:**

58 randomized and treatment-exposed patients were analyzed (28 on RAL+DRV/r and 30 on LPV/r+TVD). Greater than 10% improvement in eGFR was noted in 6 (25%) out of 24 with RAL+DRV/r and 3 (11%) of 28 with LPV/r+TVD, and the difference was not statistically significant (p=0.272, 95% CI -0.067 to 0.354). Sensitivity analyses using three other equations for eGFR showed the same results. Urinary β2 microglobulin, a sensitive marker of tenofovir tubulopathy, significantly improved with RAL+DRV/r than with LPV/r+TVD (-271 versus -64 µg/gCr, p=0.026). Per protocol analysis showed that the HIV-RNA was <50 copies/mL at week 48 in all patients of both arms (24 in RAL+DRV and 29 in LPV/r+TVD).

**Conclusions:**

Switching LPV/r+TVD to RAL+DRV/r did not significantly increase the proportion of patients who showed >10% improvement in renal function among those with relatively preserved eGFR. However, the switch improved urinary β2 microglobulin, suggesting that discontinuation of TDF might be beneficial in the long-term. RAL+DRV/r showed favorable viral efficacy in patients with suppressed viral load.

**Trial Registration:**

ClinicalTrials.gov NCT01294761 http://clinicaltrials.gov/ct2/show/NCT01294761?term=SPARE&rank=2, Umin Clinical Trials Registry UMIN000005116 http://upload.umin.ac.jp/cgi-open-bin/ctr/ctr.cgi?function=brows&action=brows&type=summary&recptno=R000006083&language=J)

##  Introduction

Tenofovir disoproxil fumarate (TDF) is one of the most widely used nucleotide reverse transcriptase inhibitors (NRTI) for patients with HIV infection, with proven efficacy and safety [1-6]. However, tenofovir is excreted by both glomerular filtration and tubular secretion, and is known to cause renal proximal tubular dysfunction. Moreover, long-term TDF use reduces glomerular filtration rate more than other NRTIs [7-10]. Although the mechanism of tenofovir-induced kidney damage is not fully understood, mitochondria toxicity, a well-known adverse event of NRTIs [11,12], in the proximal renal tubular cells is considered to be the main mechanism [13,14]. In addition to renal dysfunction, TDF also reduces bone mineral density, and both complications might lead to serious outcomes with long-term use of TDF [9,15-19]. The concurrent use of ritonavir-boosted protease inhibitors (PI/r) is a risk factor for TDF-associated nephrotoxicity, since PI/r modifies tenofovir clearance and thus increases the severity of tenofovir nephrotoxicity [20,21]. 

Clinical manifestations such as lipoatrophy and neuropathy caused by NRTI-induced mitochondria toxicity are difficult to reverse [22,23], but whether TDF nephrotoxicity is reversible after discontinuation of TDF remains unknown at present. Unfortunately, the results of few small studies that have examined this issue are contradictory [24-26]. Of note, there is no randomized controlled study that has examined the reversibility of TDF-associated nephrotoxicity. 

Recently, antiretroviral therapy (ART) not containing NRTIs (NRTI sparing regimens) has gained a wide attention, since these combinations can avoid NRTI toxicity. Despite high expectations, the results of studies on the efficacy and safety of NRTI sparing regimens for treatment-naïve patients showed dismal results. A small single arm study of CCR5 inhibitor maraviroc plus ritonavir-boosted Darunavir (DRV/r) showed a high rate of virologic failure, especially in patients with high baseline viral load of >100,000 copies/mL [27]. Raltegravir (RAL) plus unboosted atazanavir in a small randomized trial showed frequent grade 4 hyperbilirubinemia and emergence of raltegravir resistance [28]. Even the combination of RAL, a well-tolerated integrase inhibitor, and DRV/r, a protease inhibitor with high barrier to drug resistance and favorable lipid profile [29,30], showed a high prevalence of virological failure for patients with high baseline viral load in a single arm study [31]. 

At this stage, it is important to elucidate the effectiveness of NRTI sparing regimen for patients with suppressed HIV-1 viral load, because longer exposure with NRTIs tends to result in clinically overt NRTI-associated mitochondrial toxicity [22,32], and NRTI sparing regimens may avoid such long-term NRTI toxicity. Of note, the viral efficacy of NRTI-sparing regimen of RAL plus DRV/r has not been evaluated in patients with suppressed viral load [31]. 

Based on the above background, this multicenter randomized trial was conducted to elucidate 1) the reversibility of tenofovir nephrotoxicity, and 2) efficacy and safety of RAL+DRV/r for patients with suppressed viral load.

## Methods

This clinical trial was designed and reported according to the recommendations of the Consolidated Standard of Reporting Trials (CONSORT) statement [33]. The protocol for this trial and supporting CONSORT checklist are available as supporting information; see Checklist S1 and protocol S1.

### Ethics Statement

The Research Ethics Committees of Hokkaido University Hospital, Higashisaitama National Hospital, Niigata University Medical and Dental Hospital, the Institute of Medical Science, the University of Tokyo, Juntendo University School of Medicine, Shirakaba Clinic, Saku Central Hospital, Hiroshima University Hospital, Ehime University Hospital, National Hospital Organization Kyushu Medical Center, Kumamoto University Graduate School of Medical Sciences and National Center for Global Health and Medicine approved the study protocol. All patients enrolled in this study provided a written informed consent. The study was conducted according to the principles expressed in the Declaration of H e l s i n k i.

### Study Design

The SPARE trial is an on-going phase 3B, multi-center, randomized, open-label, parallel group study conducted in Japan to compare renal function and viral efficacy of NRTI-sparing regimen of RAL+DRV/r and a standard regimen of PI/r + 2NRTIs [(lopinavir/ritonavir (LPV/r) plus fixed dose of tenofovir/emtricitabine (TVD)] for 96 weeks, randomly allocated to patients on LPV/r+TVD with suppressed viral load. With one to one ratio, patients with suppressed viral load on LPV/r (800 mg/200 mg) plus fixed dose of TDF (300 mg)/emtricitabine (200 mg) were randomly assigned to either RAL (800 mg) plus DRV/r (800 mg/100 mg) or to continue LPV/r+TVD. Patient enrollment remained open between February 21, 2011 and December 2011, and the follow-up period is scheduled to end in December 2013. This report summarizes the findings after 48 weeks of treatment, including the primary endpoint. 

Randomization was stratified based on baseline body weight of 60 kg because low body weight, especially body weight of <60 kg, is an important risk for tenofovir nephrotoxicity [4,18,34]. Randomization was conducted at the data center with independent data managers, using a computer-generated randomization list prepared by a statistician with no clinical involvement in the trial.

### Study Patients

The study population included Japanese patients with HIV-1 infection, aged ≥20 years, who were on LPV/r plus TVD and with suppressed HIV-1 RNA viral load of <50 copies/ml over a period of more than 15 weeks. Patients were screened and excluded if found positive for hepatitis B surface antigen, or had history of virologic failure with regimens including protease inhibitor or integrase inhibitor, or if they were considered inappropriate for the study by the attending physicians. Candidates were also excluded if the level of alanine aminotransferase was 2.5 times the upper limit of normal, estimated glomerular filtration rate (eGFR) calculated by Cockcroft-Gault equation (CG equation) was <60 ml/min, {[(140 - age) × weight (kg)] / (serum creatinine × 72)] (× 0.85 for females)} [35], or on treatment for opportunistic infection. Actual body weight was used for the calculation of eGFR. Patients who provided written informed consent started the allocated regimens within 4 weeks of enrollment.

### Study Procedure

Visits for clinical and laboratory assessments were required within 15 weeks before registration for screening, at registration, and every 12 weeks for the duration of the study. Patients of the RAL+DRV/r arm were required to visit within 4 weeks after commencement of the allocated regimen to screen for adverse events. Baseline evaluation and evaluations at each visit covered medical history, including history of AIDS-defining illness and other comorbidities, concurrent medications, concurrent smoking, physical examination, CD4 cell count, HIV-1 RNA viral load, complete blood cell count, blood chemistries (albumin, total bilirubin, aspartate aminotransferase, alanine aminotransferase, lactate dehydrogenase, alkaline phosphatase, creatine kinase, blood urea nitrogen, serum creatinine, sodium, potassium, calcium, phosphate, triglyceride, total cholesterol, low-density lipoprotein cholesterol, high density lipoprotein cholesterol, glucose), and urine examination (urine dipstick, phosphate, creatinine, β2 microglobulin, N-acetyl-β-D-glucosaminidase (NAG), and albumin). The values of urinary β2 microglobulin, NAG, and albumin were expressed relative to urinary creatinine of 1 g/L (/g Cr). Percent tubular resorption of phosphate was calculated by the following formula: {1 – [(urine phosphate × serum creatinine) / (urine creatinine × serum phosphate)]} × 100 [36]. All data, including HIV-1 RNA viral load, were collected at each participating site and then transferred to a central data center. Grade 3 or 4 serious adverse events were reported to the independent data and safety monitoring board and analyzed for their relation to the study drugs. The grade of adverse events was classified according to the Division of AIDS Table for grading the severity of adult and pediatric events, version 2004 (URL:http://www.mtnstopshiv.org/sites/default/files/attachments/Table_for_Grading_Severity_of_Adult_Pediatric_Adverse_Events.pdf). Independent monitors visited all facilities to conduct source document verification to ensure the accuracy of all submitted data by week 48 and compliance to the protocol. All authors participated in the trial design, data analysis, and preparation of the manuscript, and vouch for the completeness and accuracy of the presented data.

### Statistical Analysis

The tested hypothesis was that more patients in the RAL+DRV/r arm will experience >10% improvement in eGFR from the baseline than patients in the LPV/r+TVD arm after switching from LPV/r+TVD to RAL+DRV/r. Sample size calculation was based on the assumption that 50% of the patients of the RAL+DRV/r arm and 10% of the patients of the LPV/r + TVD arm will experience >10% improvement in eGFR from the baseline to week 48. With a 2-sided alpha level of 0.05 and 80% power, the estimated population sample required in this study was 50 patients (25 per single arm). To account for dropouts, we planned to enroll 27 patients per one arm. The study was not fully powered for secondary analysis. Per protocol population while on the initial randomized regimen was used for the analysis of the primary endpoint.

The primary endpoint was the proportion of patients with >10% improvement in eGFR at 48 weeks from the baseline calculated with the CG equation [35]. The baseline eGFR was estimated from the average of serum creatinine measured at baseline and at screening for enrollment. eGFR at week 48 was estimated from the average of serum creatinine at weeks 36 and 48. The proportion of such patients was compared between the two arms by the Fisher exact test. The following three equations for eGFR were also used for sensitivity analysis: 1) A 3-variable equation for the Japanese set by the Japanese Society of Nephrology (JSN equation): [194 × (serum creatinine)^-1^.^094^× (age)^-0.287^× (0.739 for female patients)][37], *2*) the Modification of Diet in Renal Disease (MDRD) equation adjusted with coefficient for the Japanese [0.808 × 175 × (serum creatinine)^-1.154^ × (age)^-0.203^× (0.742 for female patients)] [37], and 3) Chronic Kidney Disease Epidemiology Collaboration (CKD-EPI) equation adjusted for the Japanese [0.813 × 141 × min (serum creatinine/κ, 1)^α^× max (serum creatinine/κ, 1) ^-1.209^ × (0.993)^age^× (1.018 for females)] (where κ is 0.7 for females and 0.9 for males, α is -0.329 for females and -0.411 for males, *min* represents the minimum of serum creatinine/κ or 1, and *max* is the maximum of serum creatinine/κ or 1) [38]. Furthermore, the percent improvement in eGFR from baseline to week 48, calculated with all four equations described above, was compared between the two arms by the Student’s t-test. Because the percent improvement in eGFR may depend on the baseline value, a correlation between the percent improvement in eGFR and the baseline value was tested, and the results showed very weak correlation (0.001<r<0.2) for all four equations for eGFR. Accordingly, the comparison of the percent improvement was conducted by the t-test as described above.

The secondary renal endpoint was changes in per protocol renal tubular markers from the baseline to week 48, and the results were compared by the Mann-Whitney test. The secondary efficacy endpoint was the proportions of patients with HIV-1 RNA <50 copies/mL at weeks 24 and 48. Data of both per protocol population and the intent-to-treat (ITT) population, comprising all randomized treatment-exposed subjects were used for the assessment of efficacy. With regard to analysis on the viral efficacy in this study, per protocol analyses were more important than ITT analyses, because some patients enrolled in the RAL+DRV/r arm were expected to develop adverse events due to switching to the new medications and subsequent discontinuation of the allocated regimen, whereas new adverse events were not likely in patients of the LPV/r+TVD arm solely by continuing the same regimen as before. Baseline parameters were compared between the two arms by the Student’s t-test for continuous variables and by either the χ^2^ test or Fisher’s exact test for categorical variables. Statistical significance was defined at two-sided *p* values <0.05. All statistical analyses were performed with The Statistical Package for Social Sciences ver. 21.0 (SPSS, Chicago, IL).

## Results

### Patient disposition and baseline characteristics

**Figure 1:**
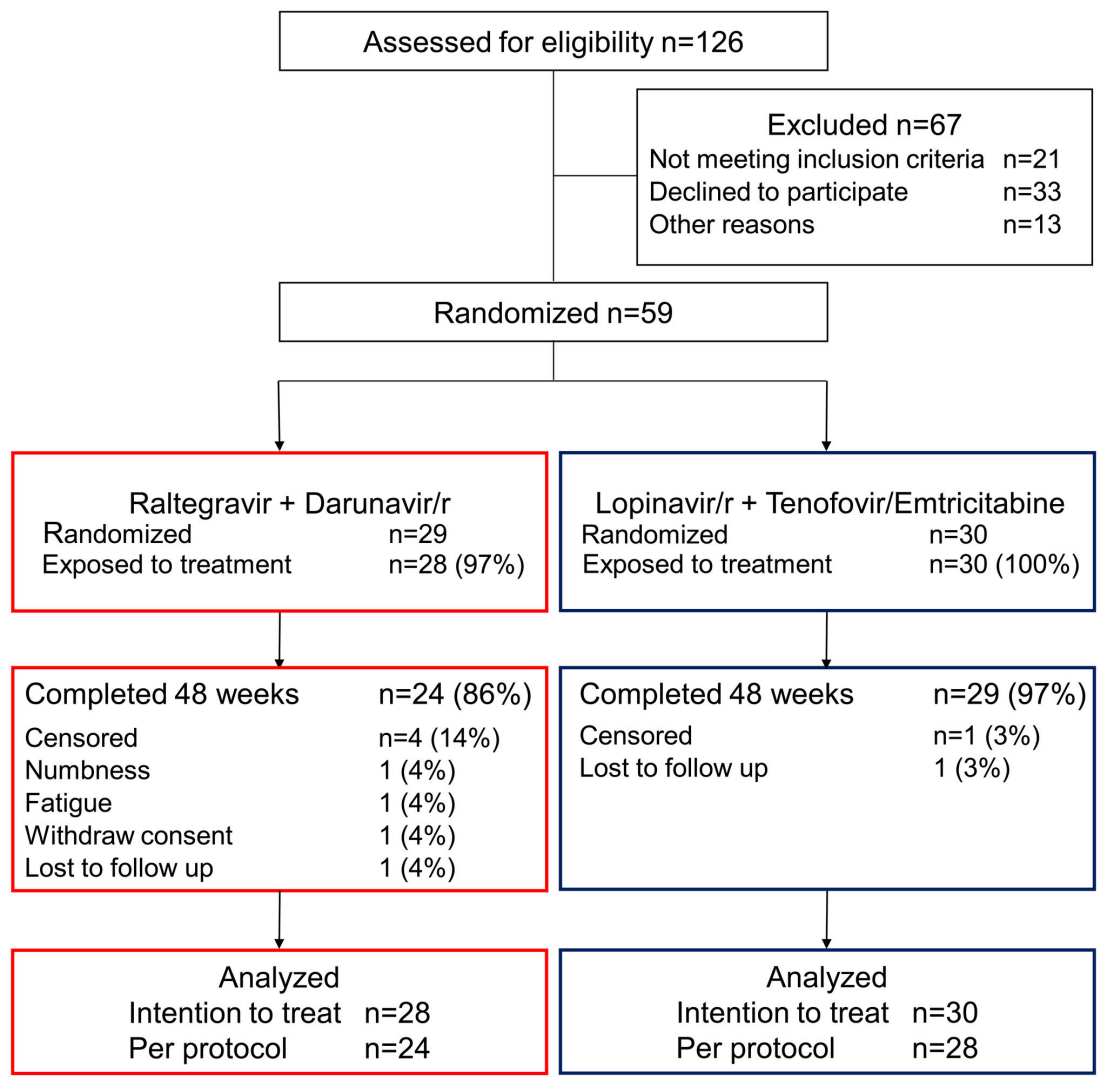
Enrollment, randomization, and disposition of patients. Darunavir/r, ritonavir-boosted darunavir; Lopinavir/r, ritonavir-boosted lopinavir.

Between February and December of 2011, 59 patients from 11 centers were enrolled in the study and randomized. Of these, 29 and 30 patients were allocated to the RAL+DRV/r and the LPV/r+TVD arm, respectively (Figure 1). One patient in the RAL+DRV/r arm withdrew consent before starting the allocated regimen, thus was excluded from the analysis. The baseline demographics and characteristics of the participating patients are listed in Table 1. Most patients were men who have sex with men, with well-maintained CD4 count. Patients of the LPV/r+TVD arm were younger (p=0.040) and had lower CD4 count (p=0.029) than those of the RAL+DRVr arm. All other major variables were similar between the two arms.

**Table 1:** Baseline characteristics of the enrolled patients.

	RAL+DRV/r (n=28)	LPV/r+TVD (n=30)	P value
Sex (male), n (%)	28 (100)	29 (97)	1.000
Age (years)^†^	44 (37-51)	39 (34-45)	0.040
CD4 count (/µl)^†^	549 (384-710)	456 (330-592)	0.029
Route of transmission (homosexual contact), n (%)	27 (96)	24 (80)	0.151
History of AIDS, n (%)	10 (36)	11 (37)	1.000
Body weight (kg)^†^	66 (59-75)	66 (59-72)	0.502
Body mass index (kg/m^2^)^†^	22 (21-25)	22.6 (19.9-24.6)	0.440
eGFR by JSN equation (ml/min/1.73 m^2^)^†^	87 (76-103)	85 (70-90)	0.356
eGFR by CG equation (ml/min)^†^	119 (88-143)	108 (89-120)	0.456
Serum creatinine (mg/dl)^†^	0.78 (0.70-0.87)	0.76 (0.67-0.83)	0.184
Urinary albumin (mg/g Cre)^†^	8 (6-27)	7 (5-12)	0.075
Urinary β2 microglobulin (μg/g Cre)^†^	452 (178-1566)	424 (204-2275)	0.234
Tubular resorption of phosphate (%)^†^	92 (87-93)	90 (86-94)	0.886
NAG (U/g Cr)^†^	6.2 (3.7-11.6)	5.2 (3.7-8.3)	0.183
Hypertension, n (%)	2 (7)	1 (3)	0.605
Dyslipidemia, n (%)	17 (61)	8 (27)	0.016
Diabetes mellitus, n (%)	0 (0)	1 (3)	1.000
Current smoking, n (%)	13 (46)	13 (43)	1.000
Hepatitis C, n (%)	0 (0)	0 (0)	N/A
Duration of tenofovir use (weeks)	163 (109-224)	124 (85-212)	0.721

Hypertension was defined by current treatment with antihypertensive agents or systolic blood pressure >140 mmHg or diastolic blood pressure >90 mmHg. Dyslipidemia was defined by current treatment with lipid-lowering agents or low-density lipoprotein cholesterol >140 mg/dl, high-density lipoprotein cholesterol <40 mg/dl, total cholesterol >240 mg/dl, or triglyceride >500 mg/dl. IQR: interquartile range, AIDS: acquired immunodeficiency syndrome, eGFR: estimated glomerular filtration rate, LDL: low-density lipoprotein, JSN: the Japanese Society of Nephrology equation [37], CG: Cockcroft-Gault equation [35]

^†^ median (interquartile range)

### Primary endpoint

At week 48, six patients (25%) out of 24 in the RAL+DRV/r arm and 3 patients (11%) out of 28 in the LPV/r+TVD arm, experienced >10% improvement in eGFR from baseline, and the difference was not statistically significant (p=0.272, 95% CI -0.067 to 0.354). Sensitivity analysis with three other equations for eGFR (JSN, CKD-EPI, and MDRD) showed the same results; no difference in the proportion of patients with improvement of >10% in eGFR was noted between the two arms (JSN equation: 4/24 in RAL+DRV/r, 3/29 in LPV/r+TVD, p=0.688, 95% CI -0.126 to 0.267) (CKD-EPI equation: 2/24 in RAL+DRV/r, 2/29 in LPV/r+TVD, p=1.000, 95% CI -0.148 to 0.197) (MDRD equation: 5/24 in RAL+DRV/r, 3/29 in LPV/r+TVD, p=0.444, 95% CI -0.093 to 0.313) (Table 2).

**Table 2:** Proportion of patients with >10% and mean percent improvement in eGFR at 48 weeks from the baseline calculated by the four equations.

	Cases with >10% increase from baseline	P value (95% CI)	Mean % improvement in eGFR from baseline	Difference in mean % improvement (95% CI) (DRV/r + RAL versus LPV/r + TDF/FTC)	P value
CG equation					
DRV/r + RAL	6/24	0.272 (-0.067 to 0.354)	5.4%	-8.7% (-18.2 to 0.8)	0.071
LPV/r + TDF/FTC	3/28		-3.3%		
JSN equation					
DRV/r + RAL	4/24	0.688 (-0.126 to 0.267)	2.5%	-1.1% (-6.9 to 4.8)	0.720
LPV/r + TDF/FTC	3/29		1.5%		
CKD-EPI equation					
DRV/r + RAL	2/24	1.000 (-0.148 to 0.197)	1.9%	-1.6% (-4.7 to 1.6)	0.323
LPV/r + TDF/FTC	2/29		1.7%		
MDRD equation					
DRV/r + RAL	5/24	0.444 (-0.093 to 0.313)	2.7%	-1.1% (-6.9 to 4.8)	0.722
LPV/r + TDF/FTC	3/29		1.7%		

DRV/r: ritonavir-boosted darunavir, RAL: raltegravir, LPV/r: ritonavir-boosted lopinavir, TDF: tenofovir, FTC: emtricitabine, CG: Cockcroft-Gault equation [35], JSN: the Japanese Society of Nephrology equation [37], CKD-EPI: Chronic Kidney Disease Epidemiology Collaboration (CKD-EPI) equation adjusted for the Japanese[38], MDRD: the Modification of Diet in Renal Disease equation adjusted with coefficient for the Japanese [37]

Additional analysis showed that the percent improvement in eGFR from the baseline to week 48 calculated using all four equations was not significantly different between the two arms [CG equation: difference in mean % improvement (DRV/r+RAL versus LPV/r+TDF/FTC) -8.7%, 95% CI -18.2 to 0.8, p=0.071] (JSN equation: -1.1%, -6.9 to 4.8, p=0.720) (CKD-EPI equation: -1.6%, 95% CI -4.7 to 1.6, p=0.323) (MDRD equation: -1.1%, 95% CI -6.9 to 4.8, p=0.722) (Table 2). Thus, this study demonstrated that switching to NRTI-sparing regimen of RAL+DRV/r did not increase the proportion of patients who showed >10% improvement in eGFR, compared to continuation of LPV/r+TVD.

### Secondary renal endpoints

**Figure 2:**
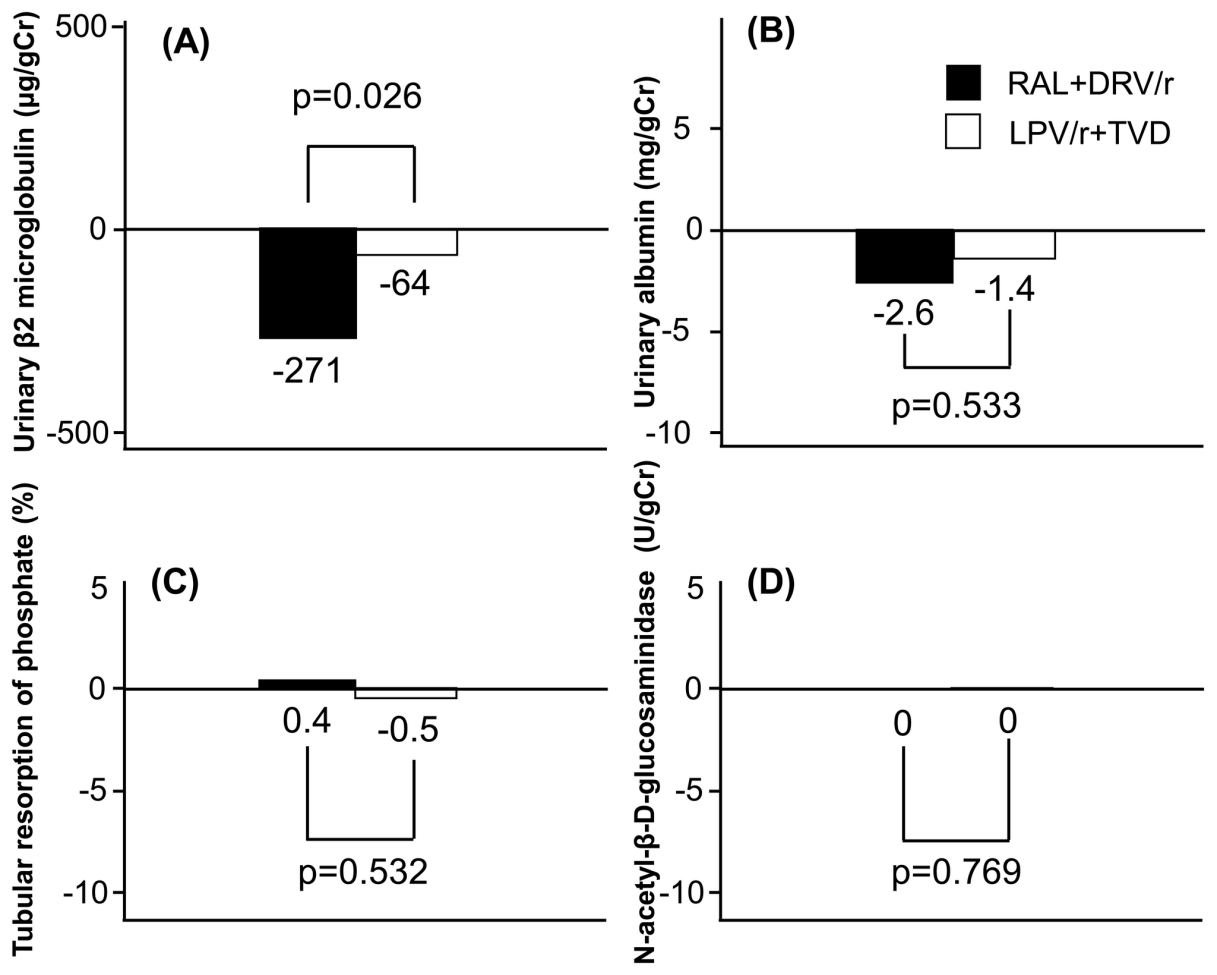
Median changes in markers of renal tubular function between baseline and 48 weeks. (A) Urinary β2 microglobulin, (B) Urinary albumin, (C) Percent tubular resorption of phosphate, (D) Urinary N-acetyl-β-D-glucosaminidase. RAL, raltegravir; DRV/r, ritonavir-boosted darunavir; LPV/r, ritonavir-boosted lopinavir; TVD, fixed dose of tenofovir/emtricitabine.

Among the four renal tubular markers used in this study, the improvement in urinary β2 microglobulin from baseline to week 48 was significantly larger in the RAL+DRV/r arm (n=23) than in the LPV/r+TVD arm (n=28) (-271 versus -64 µg/g Cr, p=0.026) (Figure 2A). However, urinary albumin, the percent tubular resorption of phosphate, and NAG showed little change from baseline, and the observed changes were not significantly different between the two arms (Figure 2B, C, D).

### Secondary efficacy endpoints

**Figure 3:**
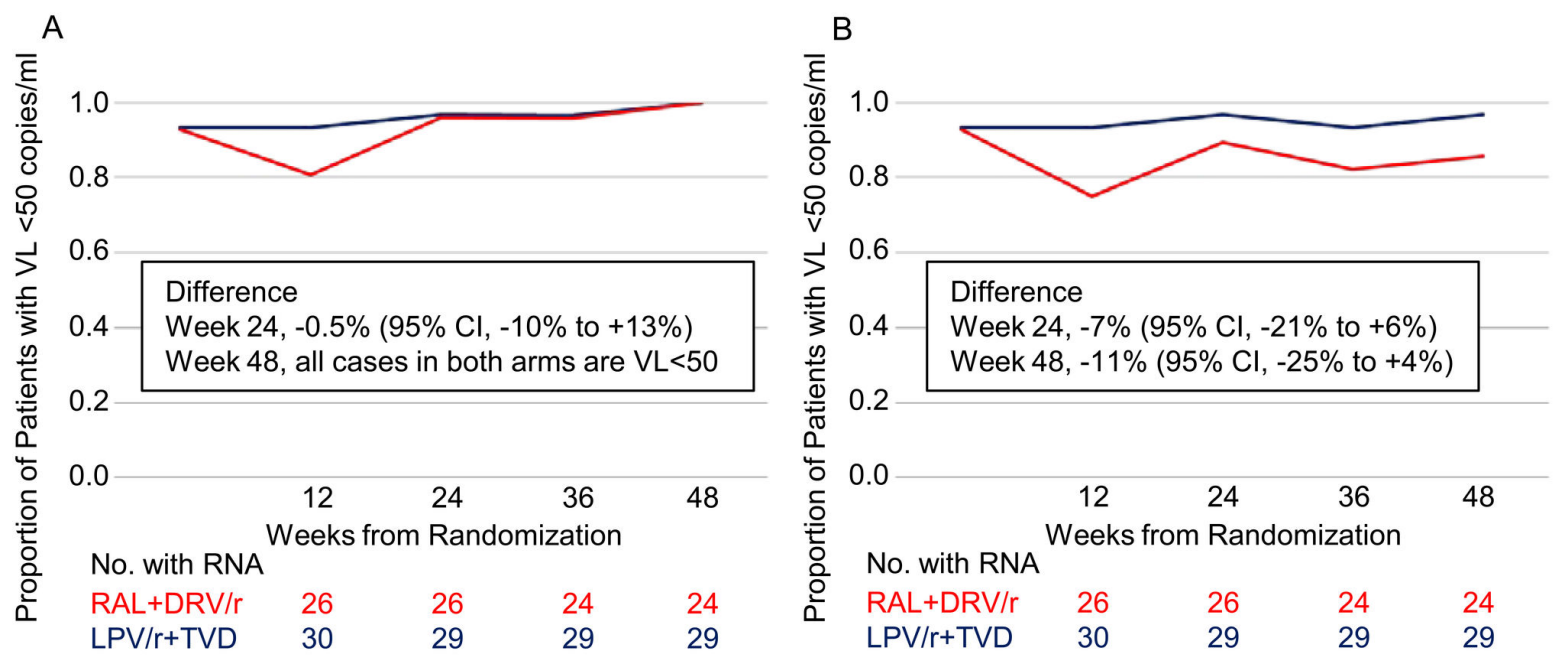
Proportion of patients with HIV RNA <50 copies/ml at 24 and 48 weeks. (A) Per protocol analysis. (B) Intention-to-treat analysis. VL, viral load; RAL, raltegravir; DRV/r, ritonavir-boosted darunavir; LPV/r, ritonavir-boosted lopinavir; TVD, fixed dose of tenofovir/emtricitabine.

Among the per protocol population, the proportion of patients with HIV RNA <50 copies/mL was 96.2% for the RAL+DRV/r arm and 96.7% for the LPV/r+TVD arm at week 24, with a difference of -0.5% (95% CI, -10% to 9%), and 100% for the both arms at week 48, with a difference of 0% (95% CI -0.1 to 0.1) (Figure 3A). ITT analysis showed that the proportion was 89.3% and 96.7% for the RAL+DRV/r and LPV/r+TVD arms, respectively, at week 24, with a difference of -7% (95% CI, -21% to 6%), and 85.7% and 96.7%, respectively, at week 48, with a difference of -11% (95% CI, -25% to 4%) (Figure 3B). There was no significant difference in viral efficacy between the two arms at weeks 24 and 48. At week 48, all patients of the RAL+DRV/r arm on the allocated regimen (n=24) had a viral load of <50 copies/mL.

### Safety and tolerability

One patient from each arm was lost to follow-up. Three patients of the RAL+DRV/r arm discontinued the allocated regimen by week 48 (one discontinued the regimen at week 4 due to weakness in the lower extremities and one at week 24 because of fatigue, which was later found to be related to acute hepatitis B infection). The other patient withdrew consent at week 24, because it was easier for him to maintain a good medication adherence with once-daily LPV/r+TVD (the regimen the patient used before enrollment). None of the patients of the LPV/r+TVD arm discontinued the allocated regimen by week 48. Thus, at week 48, 24 patients (86%) out of 28 in the RAL+DRV/r arm and 29 (97%) of 30 in the LPV/r+TVD arm, were on the allocated regimens.

The following grade 3 or 4 laboratory data or abnormal symptoms that were at least one grade higher than the baseline were encountered in this study: RAL+DRV/r arm: a rise in ALT (due to acute hepatitis B infection, n=1), and elevated LDL-cholesterol (n=3), LPV/r+TVD arm: elevated LDL-cholesterol (n=1), and hypophosphatemia (n=3). The above side effects did not lead to discontinuation of the study drugs.

## Discussion

This randomized trial elucidated the recovery of TDF-associated nephropathy after discontinuation of TDF. The results demonstrated no significant increase in the proportion of patients who showed >10% improvement in eGFR after switching to NRTI sparing regimen of RAL+DRV/r, compared to continuation of LPV/r+TVD. This finding could be due to any of the following reasons; 1) Relatively preserved baseline renal function of the enrolled patients, with a median eGFR of 86 ml/min/1.73 m^2^ (IQR 75-97, JSN equation), with only one patient with CKD stage 3 due to persistent +1 proteinuria, and no patients with stage 4 or more. Although the number of patients is relatively small, a previous pilot study of 21 patients reported improvement of eGFR (by CG equation) in most patients after switching from PI/r+TVD to PI/r+RAL in patients with proteinuria and suppressed HIV viral load [39]. Thus, improvement of eGFR after discontinuation of TDF might be more significant in patients with severe to moderately impaired renal function. Larger studies are needed to investigate this issue thoroughly. 2) Study patients had been on TDF for a long period of time at enrollment (median: 136 weeks, range 27-370 weeks, 72% were on TDF for more than 2 years), although shorter duration of TDF therapy is likely to be associated with greater eGFR improvement after discontinuation [26]. Furthermore, because TDF-induced renal dysfunction is mainly observed during the first 6 months after commencement of such therapy [18,19,40], it is possible that patients who developed severe renal dysfunction soon after starting TDF might have already discontinued TDF and therefore not included in the study.

Although the present study did not show an increase in eGFR after discontinuation of TDF, it is noteworthy that the value of urinary β2 microglobulin, a sensitive marker for TDF-induced tubulopathy [41,42], improved significantly in the RAL+DRV/r arm compared to LPV/r+TVD, even in patients with relatively preserved eGFR. It is of importance considering that proximal tubulopathy is associated with bone mineral density abnormality and possible long-term nephrotoxic effect [17,43-45]. Further large and long-term studies are needed to elucidate the long-term impact of TDF-induced tubulopathy on GFR.

With regard to the viral efficacy and safety of RAL+DRV/r, all patients in that arm who continued the allocated regimen accomplished viral suppression of <50 copies/ml at week 48 (n=24). Only one (3.6%) patient discontinued RAL+DRV/r due to a side effect possibly related to RAL+DRV/r (weakness of the lower extremities), confirming the safety of this combination. To our knowledge, this is the first study to examine the viral efficacy of RAL+DRV/r in patients with suppressed viral load. The KITE study, an industry-sponsored pilot study, examined the viral efficacy of RAL+LPV/r in patients with suppressed viral load [46]. However, LPV/r is placed as an alternative PI in the American Department of Health and Human Services Guidelines, mainly because of the higher rates of gastrointestinal side effects and hyperlipidemia compared with other PIs (URL: http://aidsinfo.nih.gov/ContentFiles/AdultandAdolescentGL.pdf). Because the number of enrolled patients is relatively small and this study does not have sufficient power to elucidate viral efficacy, further studies are needed to confirm the viral efficacy of RAL+DRV/r in patients with suppressed viral load. If the NRTI sparing regimen of RAL+DRV/r is proved to be efficacious in maintaining viral suppression in treatment-experienced patients, switching to this combination for patients with suppressed viral load should become an attractive treatment option for patients who cannot tolerate NRTI toxicity or to prevent further NRTI toxicity.

Several limitations must be acknowledged. First, as mentioned above, this trial has sufficient power for the primary endpoint only; other results should be interpreted with caution. Further larger studies are needed to confirm the improvement in urinary β2 microglobulin after switching ritonavir-boosted PI to NRTI sparing regimen of RAL+DRV/r and the viral efficacy of RAL+DRV/r in patients with suppressed viral load. Second, the enrolled patients had relatively preserved renal function. This was a study-design related issue; patients with severely impaired eGFR, the population in whom TDF nephrotoxicity can be reversible is clinically important, were excluded from the study. Based on the study design and need for randomization, patients of one arm needed to continue treatment with TDF, and it was considered ethically inappropriate to have patients with impaired renal function to continue TDF. Third, all study subjects were Japanese and almost exclusively men (mostly men who have sex with men). Further studies are needed to determine whether the findings of this study are also applicable to females, patients with different routes of transmission, and patients of different racial background.

In conclusion, this trial showed that discontinuation of LPV/r+TVD and switching to NRTI-sparing regimen of RAL+DRV/r did not result in improvement of renal function among patients with relatively preserved eGFR and suppressed HIV viral load. However, urinary β2 microglobulin, a sensitive marker of TDF-induced tubulopathy, improved after discontinuation of TDF plus ritonavir-boosted PI, suggesting switching TDF to NRTI sparing regimen might be beneficial in the long-term. RAL+DRV/r showed favorable viral efficacy and safety in patients with suppressed viral load, but further larger studies are needed to confirm the viral efficacy of this combination. 

## Supporting Information

Protocol S1 **Trial protocol.**
(DOCX)

Checklist S1 **CONSORT checklist.**
(DOC)
