# Supplementary material for: Switching Tenofovir/Emtricitabine plus Lopinavir/r to Raltegravir plus Darunavir/r in Patients with Suppressed Viral Load Did Not Result in Improvement of Renal Function but Could Sustain Viral Suppression: A Randomized Multicenter Trial
Source: PLoS One. 2013 Aug 8;8(8):e73639. doi: 10.1371/journal.pone.0073639 (PMC3738570; doi:10.1371/journal.pone.0073639)
Supplement: Protocol S1 — Trial protocol. (DOCX) [file pone.0073639.s001.docx]

**Switching to Nucleoside-Sparing Regimen of Darunavir and Raltegravir from Lopinavir/r plus Tenofovir/emtricitabine in Patients with Suppressed Viral Load:
A Randomized Open Label Multi-center Trial**

**(SPARE Trial Version 1.2)**

**Clinical Trials. gov Identifier： NCT01294761**

**Umin ID： 000005116**

Table of contents

| 1. Purpose of the trial | | 3 |
| --- | --- | --- |
| 1.1 | Main purpose of the trial | 3 |
| 1.2 | Secondary purpose of the trial | 3 |
| 2. Backgrounds and rationale of the trial | | 4 |
| 2.1 | Backgrounds | 4 |
| 2.2 | Target patients | 4 |
| 2.3 | Standard treatment for targeted patients | 4 |
| 2.4 | Trial design | 4 |
| 2.5 | Predicted benefit and risk for the patients | 5 |
| 3. Patient selection | | 6 |
| 3.1 | Eligibility | 6 |
| 3.2 | Exclusion criteria | 6 |
| 4. Protocol treatment and trial schedule | | 7 |
| 4.1 | Protocol treatment | 7 |
| 4.2 | Trial schedule | 7 |
| 4.3 | Examination schedule | 7 |
| 5. Evaluation clauses, clinical examinations, and evaluation schedule | | 8 |
| 5.1 | Baseline data and characteristics | 8 |
| 5.2 | Laboratory examination and monitoring during trial | 8 |
| 5.3 | Laboratory examination in case of discontinuation or modification of protocol-defined treatment | 9 |
| 6. Definition of examination and terms used in this trial | | 10 |
| 6.1 | CD4 cell count | 10 |
| 6.2 | HIV-1 RNA viral load | 10 |
| 6.3 | Other examinations | 10 |
| 6.4 | Visit intervals | 10 |
| 7. Registration and randomization | | 11 |
| 7.1 | Procedure of registration | 11 |
| 7.2 | Randomization | 11 |
| 8. Data collection | | 12 |
| 8.1 | CRF and due for the submission of CRF | 12 |
| 8.2 | Submission of CRFs | 12 |
| 9. Reporting adverse events | | 13 |
| 9.1 | Adverse events which need to be reported | 13 |
| 9.2 | Obligation of researcher at each center and report procedure | 13 |
| 9.3 | Obligation of principle investigator of the trial and the Trial Office | 14 |
| 9.4 | Evaluation of adverse events at the DSMB | 14 |
| 10. Definition of primary endpoint | | 15 |
| 10.1 | Primary endpoint | 15 |
| 10.2 | Definition of patients in the analysis | 15 |
| 11. Statistical consideration | | 16 |
| 11.1 | Primary endpoint | 16 |
| 11.2 | Planned number of enrolled patients | 16 |
| 11.3 | Secondary endpoints | 16 |
| 12. Ethical considerations | | 17 |
| 12.1 | Rights of the patients | 17 |
| 12.2 | Informed consent | 17 |
| 12.3 | Protection of privacy | 17 |
| 12.4 | Protocol compliance | 18 |
| 12.5 | Approval of the protocol by the IRB | 18 |
| 13. Monitoring | | 19 |
| 13.1 | Regular monitoring | 19 |
| 13.2 | On site monitoring | 19 |
| 13.3 | Protocol violation and non compliance | 19 |
| 14. Organization of the trial team (SPARE Trial Team) | | 21 |
| 14.1 | Principle investigator of the trial | 21 |
| 14.2 | Trial Office | 21 |
| 14.3 | Participating facilities and principle investigators | 21 |
| 14.4 | The Steering Committee of the clinical trial | 22 |
| 14.5 | The Data and Safety Monitoring Doard | 22 |
| 14.6 | The Data Center | 22 |
| 14.7 | Member of the Protocol Development Committee | 23 |
| 15. Reference | | 24 |
|  | |  |

1．Purpose of the trial

To elucidate *1)* the reversibility of tenofovir-associated renal dysfunction and *2)* efficacy and safety of RAL+DRV/r for patients with suppressed viral load.

1.1　Main purpose of the trial
 To examine whether more patients in the RAL+DRV/r arm will experience >10% improvement in eGFR from the baseline than patients in the LPV/r+TVD arm after switching from LPV/r+TVD to RAL+DRV/r.

1.2　Secondary purposes of the trial

1．To compare changes in per protocol renal tubular markers from the baseline to week 48 and 96.

2. To cross-sectionally compare the proportions of patients with HIV-1 RNA <50 copies/mL at weeks 48 and 96.

3. To compare changes in CD4 cell count and lipid markers.

4. Reasons for discontinuation of study drugs over 96 weeks.
5. Adverse events with grade 3 or 4 over 96 weeks

2．Backgrounds and rationale of the trial

2.1　Backgrounds

Tenofovir disoproxil fumarate (TDF) is one of the most widely used nucleotide reverse transcriptase inhibitors (NRTI) for patients with HIV infection, with proven efficacy and safety [[1-5](#_ENREF_1)]. However, tenofovir is excreted by both glomerular filtration and tubular secretion, and is known to cause renal proximal tubular dysfunction. Moreover, long-term TDF use reduces glomerular filtration rate more than other NRTIs [[6-9](#_ENREF_6)]. Although the mechanism of tenofovir-induced kidney damage is not fully understood, mitochondria toxicity, a well-known adverse event of NRTIs [[10](#_ENREF_10), [11](#_ENREF_11)], in the proximal renal tubular cells is considered to be the main cause [[12](#_ENREF_12)].

Clinical manifestations such as lipoatrophy and neuropathy caused by NRTI-induced mitochondria toxicity are difficult to reverse [[13](#_ENREF_13)], but whether TDF nephrotoxicity is reversible after discontinuation of TDF remains unknown at present.

Recently, antiretroviral therapy (ART) not containing NRTIs (NRTI sparing regimens) has gained a wide attention, since these combinations can avoid NRTI toxicity. Of note, the viral efficacy of NRTI-sparing regimen of RAL plus DRV/r has not been evaluated in patients with suppressed viral load.

Based on the above background, this multicenter randomized trial was conducted to elucidate *1)* the reversibility of tenofovir-associated renal dysfunction and *2)* efficacy and safety of RAL+DRV/r for patients with suppressed viral load.

2.2　Target patients
　　　HIV-infected patients who fulfill following conditions

(1) HIV-infected patients without history of virological failure either with protease inhibitors or raltegravir (regardless of detection of resistant virus, and treatment interruptions are permitted).

(2) Those on lopinavir/r plus tenofovir/emtricitabine (or tenofovir plus lamivudine) for more than 15 weeks.

(3) Those on lopinavir/r plus tenofovir/emtricitabine (or tenofovir plus lamivudine) and with HIV-1 viral load <50 copies/ml for more than 15 weeks.

(4) Age over 19 years

(5) Japanese

(6) Those who provide written informed consent and are willing to participate in the study

2.3　Standard treatment for targeted patients

Standard antiretroviral therapy consists of 2 NRTIs and one key drug (either protease inhibitor, integrase inhibitor, or non-NRTIs). Target patients are on LPV/r plus 2 NRTIs, thus standard therapy for those patients are to continue a current regimen.

2.4 Trial design

2.4.1　trial design

Phase 3B Randomized, open label, multi-center trial which compares renal function and viral efficacy of following 2 arms for 96 weeks;
1. LPV/r+TVD (or TDF plus 3TC) arm: Kaletra 4 tab + Truvada 1 tab (or Viread 300mg 1 tab plus Epivir 300mg 1 tab)

2. RAL+DRV/r arm: Isentress 2 tab + Prevista (400mg) 2 tab + Norvir (100mg) 1cap

2.4.2　planned sample size

54 patients

1. LPV/r+TVD arm　　 　 　27 patients

2. RAL+DRV/r arm　 　　　 27 patients

2.5　Predicted benefit and risk for the patients
2.5.1　predicted benefit

There is no benefit for the patients in LPV/r+TVD arm. Patients in RAL+DRV/r arm might benefit from this trial if patients in this arm experience more recovery of renal function than the control arm.

2.5.2　predicted risks

All medications used in this trial are widely used in daily practice. NRTI-sparing regimen such as RAL+DRV/r does not have much evidence in its efficacy and safety. However, NRTI-sparing regimen of LPV/r+RAL for treatment-naïve patients reported comparable efficacy results with patients on standard therapy [[14](#_ENREF_14)]. Protease inhibitor DRV/r used in this trial was showed to be virologically non-inferior to LPV/r among treatment-naïve or treatment-experienced patients [[15](#_ENREF_15), [16](#_ENREF_16)]. Thus RAL+DRV/r for patients with suppressed viral load is expected to be highly virologically efficacious.

3．Patient selection

Those who fulfill all of the following criteria and not applicable to any of exclusion criteria are eligible

3.1　Eligibility

Patients with HIV infection who fulfill following criteria

(1) HIV-infected patients without history of virological failure either with protease inhibitors or raltegravir (regardless of detection of resistant virus, and treatment interruptions are permitted).

(2) Those on lopinavir/r plus tenofovir/emtricitabine (or tenofovir plus lamivudine) for more than 15 weeks.

(3) Those on lopinavir/r plus tenofovir/emtricitabine (or tenofovir plus lamivudine) and with HIV-1 viral load <50 copies/ml for more than 15 weeks.

(4) Age over 19 years

(5) Japanese

(6) Those who provide written informed consent and are willing to participate in the study

3.2　Exclusion criteria

(1) Positive hepatitis B surface antigen within 15 weeks of registration.

(2) Patients with diseases which influence absorption of drugs or those who cannot swallow drugs.

(3) Patients with laboratory data described below within 15 weeks preceding and closest to the registration day

　　 　1. GPT> 2.5 times within normal limit（grade 2）

　　 　2. eGFR <60ml/min with Cockcroft-Gault equation*）

*Cockcroft-Gault eGFR=(140-age(year))×BW(kg)÷(72×sCr(mg/dl))×0.85(if female)

(4）Patients with active opportunistic infections (OIs) which require treatment (those on drugs for

primary or secondary prevention of OIs can be included).

(5) Pregnant patients or those who are giving breast feeding

(6)Those on drugs which are contraindicated to use with the study drugs

(7）Patients for whom attending physicians judge inappropriate

4．Protocol treatment and trial schedule

4.1 Protocol treatment

Enrolled patients start either one of the following protocol-defined treatments within 4 weeks after randomization

1. LPV/r+TVD (or TDF plus 3TC) arm

To continue the regimen the patient were taking before randomization:
Kaletra 4 tab + Truvada 1 tab (or Viread 300mg 1 tab plus Epivir 300mg 1 tab)

2. RAL+DRV/r arm

To change the regimen to:

Isentress 2 tab + Prevista (400mg) 2 tab + Norvir (100mg) 1cap

　4.2　Trial schedule

　　　　1.　Screening to registration: within 15 weeks

　　　　2.　Randomization to the start of treatment: within 4 weeks

　　　　3.　The day of randomization is defined as Day 1

4.3 Examination schedule

Patients in RAL+DRV/r arm are required to visit within 4 weeks after starting the allocated regimen to monitor adverse events. Patients in both arms are required to visit every 12 weeks to conduct protocol-defined observation and examination. At 48 weeks, primary endpoint and secondary endpoints are evaluated. After 48 weeks, enrolled patients are required to visit every 12 weeks to conduct protocol-defined observation and examination until week 96.

5．Evaluation clauses, clinical examinations, and evaluation schedule

　5.1 Baseline data and characteristics （Day1）

　　　Baseline data are collected within 15 weeks preceding and including the day of registration

1）patient characteristics：　history of AIDS, complications, past history of other diseases, concomitant medications, current smoking, HIV-related variables (date of starting ART, date of starting TDF, date of starting protease inhibitor)

2）physical examinations：　body weight, height

3）laboratory data:

-CD4 cell count

-HIV-1 RNA viral load

-white blood cell count, hemoglobin, platelet

-serum albumin, total bilirubin, aspartate transaminase, alanine aminotransferase, lactate dehydrogenase, alkaline phosphatase, creatine kinase, blood urea nitrogen, serum creatinine, sodium, potassium, calcium, phosphate, total cholesterol, triglyceride, low density lipoprotein cholesterol, high density lipoprotein cholesterol, glucose

-urine (dipstick test)

-urine phosphate, urine creatinine, urine β2 microglobulin, urine N-acetyl-β-D-glucosaminidase, urinary albumin

5.2　Laboratory examination and monitoring during trial

5.2.1　Laboratory examination required to conduct for patients in both arms every 12 weeks from randomization to week 96

1）physical examinations：　body weight, symptom, abnormal physical findings, newly emerged complications

2) drugs: adherence, concomitant medications

3）laboratory data (blood should be principally taken after meal)

-CD4 cell count

-HIV-1 RNA viral load

-white blood cell count, hemoglobin, platelet

-serum albumin, total bilirubin, aspartate transaminase, alanine aminotransferase, lactate dehydrogenase, alkaline phosphatase, creatine kinase, blood urea nitrogen, serum creatinine, sodium, potassium, calcium, phosphate, total cholesterol, triglyceride, low density lipoprotein cholesterol, high density lipoprotein cholesterol, glucose

-urine (dipstick test)

-urine phosphate, urine creatinine, urine β2 microglobulin, urine N-acetyl-β-D-glucosaminidase, urinary albumin

5.2.2　Laboratory examination required to conduct for patients in RAL+DRV/r arm within 4 weeks from starting allocated regimens

1）physical examinations：　body weight, symptom, abnormal physical findings, newly emerged complications

2) drugs: adherence, concomitant medications

3）laboratory data (blood should be principally taken after meal)

-CD4 cell count (can be omitted)

-HIV-1 RNA viral load (can be omitted)

-white blood cell count, hemoglobin, platelet

-serum albumin, total bilirubin, aspartate transaminase, alanine aminotransferase, lactate dehydrogenase, alkaline phosphatase, creatine kinase, blood urea nitrogen, serum creatinine, sodium, potassium, calcium, phosphate, total cholesterol, triglyceride, low density lipoprotein cholesterol, high density lipoprotein cholesterol, glucose

-urine (dipstick test)

-urine phosphate, urine creatinine, urine β2 microglobulin, urine N-acetyl-β-D-glucosaminidase

5.3　Laboratory examination in case of discontinuation or modification of protocol-defined treatment

In case of discontinuation or modification of protocol-defined treatment due to adverse event, patients are required to conduct following examination at discontinuation and within 4 weeks from discontinuation if possible:

1）physical examinations：　body weight, symptom, abnormal physical findings, newly emerged complications

2) drugs: adherence, concomitant medications

3）laboratory data (blood should be principally taken after meal)

-CD4 cell count (can be omitted)

-HIV-1 RNA viral load (can be omitted)

-white blood cell count, hemoglobin, platelet

-serum albumin, total bilirubin, aspartate transaminase, alanine aminotransferase, lactate dehydrogenase, alkaline phosphatase, creatine kinase, blood urea nitrogen, serum creatinine, sodium, potassium, calcium, phosphate, total cholesterol, triglyceride, low density lipoprotein cholesterol, high density lipoprotein cholesterol, glucose

-urine (dipstick test)

-urine phosphate, urine creatinine, urine β2 microglobulin, urine N-acetyl-β-D-glucosaminidase, urinary albumin

6．Definition of examination and terms used in this trial

6.1　CD4 cell count

Flow cytometry methods are used. This is conducted in each participating center or laboratories

6.2　HIV-1 RNA viral load

Roche Cobas Taqman assay version 1.5 was used until November 2011, and after that version 2.0 was used to measure HIV-1 RNA.

6.3　Other examinations

Laboratory tests are conducted in each participating center and laboratories.

6.4　Visit intervals

Day of randomization is defined as Day1. In principle, patients allocated to RAL+DRV/r arm need to visit within 4 weeks after randomization. Patients in both arms are required to visit every 12 weeks to conduct protocol-defined observation and examinations. Week 4 in this trial is defined as the period including before and after 2 weeks from 4 weeks after the day of randomization. Week 48 is defined as the period including before and after 6 weeks from 48 weeks after the day of randomization. Week 96 is defined as the period including before and after 6 weeks from 96 weeks after the day of randomization. Similarly, Week 12, Week 24, Week 36, Week 60, Week 72, Week 84 are defined as the period including before and after 6 weeks of the weeks from day of randomization.

7．Registration and randomization

7.1 Procedure of registration

Each participating center confirms that a candidate for the trial fulfills all the eligibility criteria and do not fulfill any exclusion criteria. The participating center submits CRF-1 to the data center. The data center confirms that the patient is eligible, and registers and randomizes the patient.

7.2　Randomization

Randomization was stratified based on baseline body weight of 60 kg because low body weight, especially body weight of <60 kg, is an important risk for tenofovir nephrotoxicity (unpublished data). Randomization was conducted at the data center with independent data managers, using a computer-generated randomization list prepared by a statistician with no clinical involvement in the trial.

8．Data collection

8.1　CRF and due for the submission of CRF

（1） Case Report Form (CRF） in this trial and submission due for the CRFs are following:

| Registration form | CRF-1 | Within 15 weeks from obtaining informed consent |
| --- | --- | --- |
| Regular CRF | CRF-2 | Within 4 weeks after visit |
| Discontinuation form | CRF-3 | Within 15 days from discontinuation of study drugs |
| Follow up for discontinuation | CRF-3A | within 4 weeks after visit |
| Adverse event form | CRF-4 | Within 24 hours for the 1st emergency report, within 15 day for 2nd emergency report and other adverse event |
| Death report | CRF-5 | Within 15 days after death |

8.2　Submission of CRFs

All CRFs needs to be submitted to the data center

　　　　　The Data Center:　AIDS Clinical Center, National Center for Global Health and Medicine

FAX: 03-5273-6483

9．Reporting adverse event

Severe adverse events or unexpected adverse events need to be reported to the Data Center with CRF-4. The Data Center immediately conveys the information to the principle investigator, the Trial Office, data and safety monitoring board. The grade of adverse event is classified according to the DIVISION OF AIDS TABLE FOR GRADING THE SEVERITY OF ADULT AND PEDIATRIC ADVERSE EVENTS (publish date: December, 2004). Report of adverse events to a director of each participating center and the Japanese Ministry of Health and Welfare are according to the rule of each center, and are conducted by the investigator at each center.

9.1　Adverse events which need to be reported

9.1.1 Emergency adverse events （CRF-4）

Adverse events which fulfills either one of following criteria

　　　　　 1）Any death within 30 days after starting protocol-defined treatment.

2）Unexpected grade 4 adverse events

9.1.2　adverse events need to be reported（CRF-4）

1）Expected grade 4 adverse events

2）unexpected grade 2 or 3 adverse events

9.1.3　Death（CRF-5）

All death cases during study period need to be reported

9.2　Obligation of researcher at each center and report procedure

9.2.1　Emergency report of adverse events

At the time of emergency adverse events, attending physicians report principle investigator at each center. When an attending physician cannot contact principle investigator at the center, attending physician needs to report the case on behalf of principle investigator.

1st emergency report: principle investigator at each participating center need to report the adverse event within 24 hours from occurrence with CRF-4 (first report) and to make a phone call

TEL: 03-3202-7181

FAX: 03-5273-6483　AIDS Clinical Center Data Center

2nd emergency report：the principle investigator at each participating center needs to report with CRF-4 (second report) within 15 days from occurrence of adverse events.

9.2.2　report of adverse events

Principle investigator at each participating center needs to report protocol-defined adverse events within 15 days after occurrence of adverse events with CRF-4

　　　　9.2.3　Death report

　　　　　 Principle investigator at each participating center needs to report death case with CRF-5 within 15

days from occurrence of death

9.3　Obligation of principle investigator of the trial and Trial Office

9.3.1 Judgment for halting of registration and emergency notice to each participating center

Principle investigator of the trial and the Trial Office make a judgement on the needs for halting of registration or emergency notice to each participating center.

9.3.2 Report to the independent data and safety monitoring board (DSMB)

When the principle investigator of the trial judges that the adverse events reported apply to the clause 9.1 “Adverse events which need to be reported”, he needs to report to the DSMB with a written form within 15 days after receiving a report.

9.4　Evaluation of adverse events at the DSMB

The DSMB evaluates the report from the principle investigator of the trial, and makes a recommendation for continuation or discontinuation of registration and a need for protocol revision. The result of evaluation and recommendations are send to the principle investigator of the trial and the trial office.

10．Definition of primary endpoint

10.1　Primary endpoint

The primary endpoint was the proportion of patients with >10% improvement in eGFR at 48 weeks from the baseline calculated with the Cockcroft-Gault equation.

10.2　Definition of patients in the analysis

In ITT analysis, all enrolled patients who were randomized and exposed to the treatment are included. For per protocol analysis, a number of patients on the allocated treatment at each time points are used.

10.2.1　ITT population

All enrolled patients who were randomized and exposed to the treatment according to the procedure described in “7.1 procedure of registration”.

10.2.2　Per protocol population

A number of patients on the allocated treatment at each time points are defined as per protocol population.

11．Statistical consideration

11.1　Primary endpoint

The tested hypothesis was that more patients in the RAL+DRV/r arm will experience >10% improvement in eGFR from the baseline than patients in the LPV/r+TVD arm after switching from LPV/r+TVD to RAL+DRV/r. The primary endpoint was the proportion of patients with >10% improvement in eGFR at 48 weeks from the baseline calculated with the Cockcroft-Gault equation. The proportion of such patients was compared between the two arms with the χ^2^ test. Per protocol population while on the initial randomized regimen was used for the analysis of the primary endpoint.

- 1. Planned number of enrolled patients

Sample size calculation was based on the assumption that 50% of the patients of the RAL+DRV/r arm and 10% of the patients of the LPV/r + TVD arm will experience >10% improvement in eGFR from the baseline to week 48. With a 2-sided alpha level of 0.05 and 80% power, the estimated population sample required in this study was 50 patients (25 per single arm). To account for dropouts, we planned to enroll 27 patients per one arm. The study was not fully powered for secondary analysis.

11.3　Secondary endpoints

11.3.1　Secondary efficacy endpoints

-To cross-sectionally compare the proportion of the patients with HIV-1 RNA <50 copies/ml at week 48 and 96.

11.3.2　Secondary renal endpoints

- The secondary renal endpoint was changes in per protocol renal tubular markers (β2 microglobulin, N-acetyl-β-D-glucosaminidase, albumin, percent tubular resorption of phosphate) from the baseline to week 48 and week 96, and the results were compared by the Student’s t-test.

11.4.3 Other endpoints

-To compare changes in per protocol CD4 cell count, lipid markers.

12．Ethical considerations

12.1　Right of the patients

All investigators of this trial follow the Helsinki declaration and the guideline for ethical consideration for clinical trial by the Japanese ministry of health and welfare: <http://www.mhlw.go.jp/general/seido/kousei/i-kenkyu/index.html>

12.2　Informed consent

12.2.1　Informed consent to the patients

Prior to the registration, an attending physician hand the consent form which was approved by IRB of the facility and explain following clauses.

1）Backgrounds and rationale of trial

2）Purpose of the trial and planned number of enrolled patients

3）Assigned treatment

4）Visit intervals

5）Duration of the trial

6）Expected adverse events

7）Cost

8）Expected treatment when declining to participate in the study

9) Procedures to discontinue participation of the trial

10）Procedures to any harms in health during trial

11) Protection of the privacy

12）Safety procedure for the patients

13) Freedom for asking questions

12.2.2　Informed consent with a form and preservation of informed consent forms

When consent is gained the patient and attending physician sign the consent form with the date. One copy of the consent will be given to the patient and the original form will be preserved in the patient file with CRFs

12.3　Protection of privacy

Each registered patient is deidentified with the registration number and only the registration number is reported to the data center.

12.4　Protocol compliance

All investigators participating in this trial comply with the protocol.

12.5　Approval of the protocol by the IRB

12.5.1　 Approval of the protocol by the IRB

Each participating center needs to obtain approval for the protocol and informed consent form by the IRB at the center

13．Monitoring

13.1　Regular monitoring
Central monitoring is conducted annually to monitor that the trial is safely conducted according to the protocol and the data are precisely collected. The Data Center submit the regular monitoring reports to the DSMB, the principle investigator, and the Trial Office.

13.1.1　Clauses of monitoring
1) The number of enrolled patients
2) Patients in trial, patients who discontinued allocated regimens and reasons for discontinuation.

3) Serious adverse events

4) Adverse events
5) Violation of the protocol

6) Other issues in trial processing and safety

13.2　On site monitoring

The annual site visits are conducted to maintain scientific and ethical quality of the trial. On site monitoring are conducted to at least 5 participating centers and the data in CRFs and original data are cross checked. All participating sites are to be visited by the end of the trial. If annual monitoring finds more than 2 facilities with more than one case of protocol violation, all participating centers are to be visited for on site monitoring in that year. On site monitoring are to be conducted by the independent research coordinators.

13.2.1　Clauses of on site monitoring

- Confirmation of approval letter by IRB of the facility
- Confirmation of informed consent form
- Cross checking CRF and original data
- Monitor adverse events
- Monitor protocol violation
- Confirm that forms are preserved in the safe place

13.3 Protocol violation and non compliance
Protocol violation is defined as cases to whom drug allocation and laboratory examination were not conducted according to the protocol. As a general rule, a conduct of non compliance which was predefined by the Data Center, the principle investigator, and the Trial Office was recorded in the monitoring reports at the time of on site visits. A conduct of non compliance is classified into either “protocol violation” or “a conduct of non compliance”

1) Protocol violation
As a general rule, a conduct of non compliance from the protocol which fulfills more than one of following clauses is defined as “protocol violation”
　-affects the endpoint of the trial
　-intentional or systemic
　-affects safety of the enrolled patients or the extent of violation is substantial
　-clinically inappropriate
　Protocol violations are described in the publishing paper as principle.

2) Non compliance
a conduct of non compliance which is not included in 1) or 3). If a particular conduct of non compliance is often observed, that will be described in the publishing paper.

3) Acceptable non compliance
Acceptable conducts of non compliance were predefined by the Data Center, the principle investigator, and the Trial Office. As a general rule, a conduct of non compliance which suits following criteria are defined as “acceptable non compliance”
　- do not affect the endpoint of the trial
　- not intentional
　- degree of non compliance is subtle.

14．Organization of the trial team (SPARE Trial Team)

14.1　Principle Investigator of the trial

Shinichi OKA

AIDS Clinical Center, National Center for Global Health and Medicine

Toyama 1-21-1, Shinjuku, Tokyo, Japan 162-8655

TEL: 03-3202-7181 FAX: 03-3208-4244

E-mail：oka@acc.ncgm.go.jp

14.2　Trial Office

Takeshi NISHIJIMA

AIDS Clinical Center, National Center for Global Health and Medicine

Toyama 1-21-1, Shinjuku, Tokyo, Japan 162-8655

TEL: 03-3202-7181 FAX: 03-3208-4244

E-mail：tnishiji@acc.ncgm.go.jp

14.3　Participating facilities and principle investigators

| CODE | Name of the facilities | Principle Investigators |
| --- | --- | --- |
| ACC | National Center for Global Health and Medicine | Kunihisa TSUKADA |
| HKU | Hokkaido University Hospital | Tomoyuki ENDO |
| NGU | Niigata University Medical and Dental Hospital | Yoshinari TANABE |
| HSU | Hiroshima University Hospital | Teruhisa FUJII |
| KMC | National Hospital Organization Kyushu Medical Center | Masahiro YAMAMOTO |
| HSH | Higashisaitama National Hospital | Masahide HORIBA |
| IMS | Research Hospital of the Institute of Medical Science, The University of Tokyo | Michiko KOGA |
| JNU | Juntendo University School of Medicine | Toshio NAITO |
| SRS | Shirakaba Clinic | Ichiro ITODA |
| SKH | Saku Central Hospital | Masanori TEI |
| EHU | Ehime University Hospital | Kiyonori TAKADA |
| KMU | Kumamoto University | Toshikazu MIYAKAWA |

14.4　 The Steering Committee of the clinical trial

Chairperson of the Steering Committee

Shinichi OKA

AIDS Clinical Center, National Center for Global Health and Medicine

Toyama 1-21-1, Shinjuku, Tokyo, Japan 162-8655

TEL: 03-3202-7181 FAX: 0.-3208-4244

E-mail：oka@acc.ncgm.go.jp

Member of the Steering Committee

Yoshimi KIKUCHI National Center for Global Health and Medicine

Hiroyuki GATANAGA National Center for Global Health and Medicine

Kunihisa TSUKADA National Center for Global Health and Medicine

Masayuki CHIDA　 National Center for Global Health and Medicine

14.5　 The Data and Safety Monitoring Board

Chairman of the Data and Safety Monitoring Board

Hiroaki MITSUYA

Departments of Infectious Diseases and Hematology, Kumamoto University Graduate School of Medical Sciences,

Members of the Data and Safety Monitoring Board

Satoshi KIMURA Tokyo Teishin Hospital

Takuro SHIMBO National Center for Global Health and Medicine

Yoshinari TANABE 　 Niigata University Medical and Dental Hospital

Masayuki CHIDA

National Center for Global Health and Medicine, Division of Pharmacy

14.6 The Data Center

Division of Medical Informatics, Department of Clinical Research and Informatics, National Center for Global Health and Medicine

AIDS Clinical Center, National Center for Global Health and Medicine

Toyama 1-21-1, Shinjuku, Tokyo, Japan 162-8655

TEL&FAX: 03-5273-6483

Director of the Data Center and in charge of statistical analysis

Takuro SHIMBO　　

Division of Medical Informatics, Department of Clinical Research and Informatics, National Center for Global Health and Medicine

Statistical manager

Naoki ISHIZUKA

Division of Medical Informatics, Department of Clinical Research and Informatics, National Center for Global Health and Medicine

Data managers

Michiyo ISHISAKA, Mikiko OGATA, Misao TAKANO, Fumihide KANAYA

AIDS Clinical Center, National Center for Global Health and Medicine

Development of CRFs

Michiyo ISHISAKA, Mikiko OGATA, Misao TAKANO

AIDS Clinical Center, National Center for Global Health and Medicine

Site visit manager

Akiko NAKANO

AIDS Clinical Center, National Center for Global Health and Medicine

14.7　Member of the Protocol Development Committee

Shinichi OKA, Yoshimi KIKUCHI, Hiroyuki GATANAGA, Kunihisa TSUKADA, Junko TANUMA, Takeshi NISHIJIMA, Michiyo ISHISAKA, Misao TAKANO, Mikiko OGATA, and Fumihide KANAYA

AIDS Clinical Center, National Center for Global Health and Medicine

Masayuki CHIDA, Junichi MASUDA, Mai NAKAMURA

Division of Pharmacy, National Center for Global Health and Medicine

15．Reference

1. Gallant JE, DeJesus E, Arribas JR, Pozniak AL, Gazzard B, Campo RE*, et al.* Tenofovir DF, emtricitabine, and efavirenz vs. zidovudine, lamivudine, and efavirenz for HIV. *N Engl J Med* 2006,**354**:251-260.

2. Gallant JE, Staszewski S, Pozniak AL, DeJesus E, Suleiman JM, Miller MD*, et al.* Efficacy and safety of tenofovir DF vs stavudine in combination therapy in antiretroviral-naive patients: a 3-year randomized trial. *JAMA* 2004,**292**:191-201.

3. Izzedine H, Hulot JS, Vittecoq D, Gallant JE, Staszewski S, Launay-Vacher V*, et al.* Long-term renal safety of tenofovir disoproxil fumarate in antiretroviral-naive HIV-1-infected patients. Data from a double-blind randomized active-controlled multicentre study. *Nephrol Dial Transplant* 2005,**20**:743-746.

4. Nelson MR, Katlama C, Montaner JS, Cooper DA, Gazzard B, Clotet B*, et al.* The safety of tenofovir disoproxil fumarate for the treatment of HIV infection in adults: the first 4 years. *AIDS* 2007,**21**:1273-1281.

5. Arribas JR, Pozniak AL, Gallant JE, Dejesus E, Gazzard B, Campo RE*, et al.* Tenofovir disoproxil fumarate, emtricitabine, and efavirenz compared with zidovudine/lamivudine and efavirenz in treatment-naive patients: 144-week analysis. *J Acquir Immune Defic Syndr* 2008,**47**:74-78.

6. Kinai E, Hanabusa H. Progressive renal tubular dysfunction associated with long-term use of tenofovir DF. *AIDS Res Hum Retroviruses* 2009,**25**:387-394.

7. Winston A, Amin J, Mallon P, Marriott D, Carr A, Cooper DA*, et al.* Minor changes in calculated creatinine clearance and anion-gap are associated with tenofovir disoproxil fumarate-containing highly active antiretroviral therapy. *HIV Med* 2006,**7**:105-111.

8. Gallant JE, Winston JA, DeJesus E, Pozniak AL, Chen SS, Cheng AK*, et al.* The 3-year renal safety of a tenofovir disoproxil fumarate vs. a thymidine analogue-containing regimen in antiretroviral-naive patients. *AIDS* 2008,**22**:2155-2163.

9. Fux CA, Simcock M, Wolbers M, Bucher HC, Hirschel B, Opravil M*, et al.* Tenofovir use is associated with a reduction in calculated glomerular filtration rates in the Swiss HIV Cohort Study. *Antivir Ther* 2007,**12**:1165-1173.

10. Nolan D, Mallal S. Complications associated with NRTI therapy: update on clinical features and possible pathogenic mechanisms. *Antivir Ther* 2004,**9**:849-863.

11. Lewis W, Day BJ, Copeland WC. Mitochondrial toxicity of NRTI antiviral drugs: an integrated cellular perspective. *Nat Rev Drug Discov* 2003,**2**:812-822.

12. Kohler JJ, Hosseini SH, Hoying-Brandt A, Green E, Johnson DM, Russ R*, et al.* Tenofovir renal toxicity targets mitochondria of renal proximal tubules. *Lab Invest* 2009,**89**:513-519.

13. Moyle G. Clinical manifestations and management of antiretroviral nucleoside analog-related mitochondrial toxicity. *Clin Ther* 2000,**22**:911-936; discussion 898.

14. Reynes J, Lawal A, Pulido F, Soto-Malave R, Gathe J, Tian M*, et al.* Examination of noninferiority, safety, and tolerability of lopinavir/ritonavir and raltegravir compared with lopinavir/ritonavir and tenofovir/ emtricitabine in antiretroviral-naive subjects: the progress study, 48-week results. *HIV Clin Trials* 2011,**12**:255-267.

15. Madruga JV, Berger D, McMurchie M, Suter F, Banhegyi D, Ruxrungtham K*, et al.* Efficacy and safety of darunavir-ritonavir compared with that of lopinavir-ritonavir at 48 weeks in treatment-experienced, HIV-infected patients in TITAN: a randomised controlled phase III trial. *Lancet* 2007,**370**:49-58.

16. Mills AM, Nelson M, Jayaweera D, Ruxrungtham K, Cassetti I, Girard PM*, et al.* Once-daily darunavir/ritonavir vs. lopinavir/ritonavir in treatment-naive, HIV-1-infected patients: 96-week analysis. *AIDS* 2009,**23**:1679-1688.
